# Overcoming Normalcy Bias in Acute Myocardial Infarction: A Case Report of Generative AI as a Behavioral Catalyst for Emergency Care Seeking

**DOI:** 10.7759/cureus.101199

**Published:** 2026-01-09

**Authors:** Osamu Ikeda, Naofumi Oyamada, Masashi Hayashi

**Affiliations:** 1 Faculty of Human Development and Education, Kyoto Tachibana University, Kyoto, JPN; 2 Department of Cardiology, Japanese Red Cross Otsu Hospital, Otsu, JPN

**Keywords:** acute myocardial infarction, artificial intelligence, behavioral catalyst, generative ai, large language models, normalcy bias, patient decision making, percutaneous coronary intervention, pre-hospital delay, stemi

## Abstract

Pre-hospital delay remains a major determinant of outcomes in acute myocardial infarction (AMI), with normalcy bias playing a central role in patients’ failure to interpret symptoms as signals of serious illness. This case report examines the role of generative artificial intelligence (AI) not as a diagnostic instrument but as a behavioral catalyst that prompted timely emergency care seeking. A man in his early sixties presented with chest discomfort, neck radiation, bilateral lower molar pain, diaphoresis, and cold extremities. Although these symptoms are medically typical of AMI, myocardial infarction was not part of the patient’s immediate cognitive framework, and they were initially interpreted as dental discomfort or nonspecific physical fatigue. After consulting a publicly available generative AI system that issued a clear imperative to contact emergency medical services, the patient activated emergency care. He was subsequently diagnosed with inferior ST-elevation myocardial infarction due to right coronary artery occlusion and underwent successful emergency percutaneous coronary intervention. This case suggests that AI-generated language can mitigate normalcy bias and accelerate patient decision-making in acute medical settings without functioning as a diagnostic tool.

## Introduction

Acute myocardial infarction (AMI) remains a leading cause of morbidity and mortality worldwide [1,2]. While advances in reperfusion therapy and in-hospital systems of care have substantially improved outcomes, patient-related pre-hospital delay continues to represent a critical bottleneck [3,4]. Psychological factors, particularly normalcy bias, the tendency to downplay threat signals and reinterpret them as benign, play a substantial role in delayed help-seeking behavior [5].

Public health education has traditionally emphasized recognition of “typical” symptoms, such as chest pain radiating to the arm or jaw. However, awareness alone does not reliably translate into timely action. Under conditions of stress and uncertainty, individuals often fail to apply abstract medical knowledge to their own subjective experiences, rationalizing symptoms as minor ailments such as fatigue, gastrointestinal discomfort, or dental pain. Indeed, orofacial pain can sometimes be the sole symptom of cardiac ischemia, leading to dangerous misinterpretation [6].

Generative artificial intelligence (AI) has primarily been evaluated for its diagnostic accuracy and information retrieval capabilities [7,8]. Its potential role in shaping patient behavior at the moment of symptom onset, however, remains underexplored. We report a case in which AI-generated language directly preceded a decisive behavioral shift, prompting emergency care seeking in a patient experiencing normalcy bias.

## Case presentation

Patient information

The patient was a man in his early sixties with a history of hypertension, chronic kidney disease (stage G3a), dyslipidemia, and hyperuricemia. He was adherent to prescribed medications. He was not a medical professional.

Symptom onset and decision-making process

In late August 2025, at approximately 11:40 AM, the patient developed chest discomfort with neck radiation, bilateral lower molar pain, diaphoresis, and cold extremities. At symptom onset, myocardial infarction was not part of the patient’s cognitive interpretation. As a non-medical individual, he interpreted these sensations as dental discomfort or nonspecific physical fatigue. The patient measured his vital signs at home, which showed a heart rate of approximately 100 beats per minute and a systolic blood pressure of approximately 110 mmHg. Because these values did not appear markedly abnormal to him, he perceived the situation as noncritical and hesitated to seek medical attention. This hesitation was further reinforced by normalcy bias and plan continuation bias. Additionally, nearby cardiology clinics were closed that day, which contributed to his reluctance to seek immediate medical evaluation.

Incident and AI consultation

Because the toothache and discomfort persisted, the patient entered his subjective symptoms (“chest and neck discomfort, toothache, cold sweat”) into a commercially available generative AI system. Rather than providing a list of differential diagnoses, the AI issued a concise and imperative recommendation advising immediate contact with emergency medical services. The patient repeated the query twice, and the AI consistently reiterated the same urgent instruction. This externally generated directive preceded a decisive behavioral shift. At 11:51 AM, approximately 11 minutes after symptom onset, the patient instructed his family to contact emergency medical services. While waiting for the ambulance, he instructed his family to prepare the patient registration card for his regular hospital and to bring his mobile phone charger. He also directed the arriving paramedics to use an elevator suitable for stretcher transport.

Emergency care, diagnosis, and treatment

**Figure 1:**
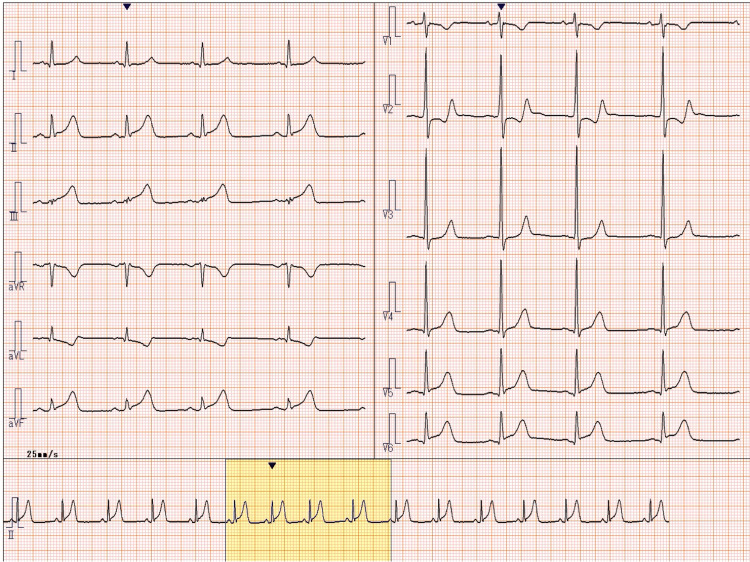
Electrocardiogram obtained on admission demonstrating ST-segment elevation in the inferior leads Electrocardiogram obtained on admission (August 27, 2025, 12:16 PM) demonstrating ST-segment elevation in leads II, III, and aVF, consistent with acute inferior ST-elevation myocardial infarction. aVF: arteriovenous fistula.

**Figure 2:**
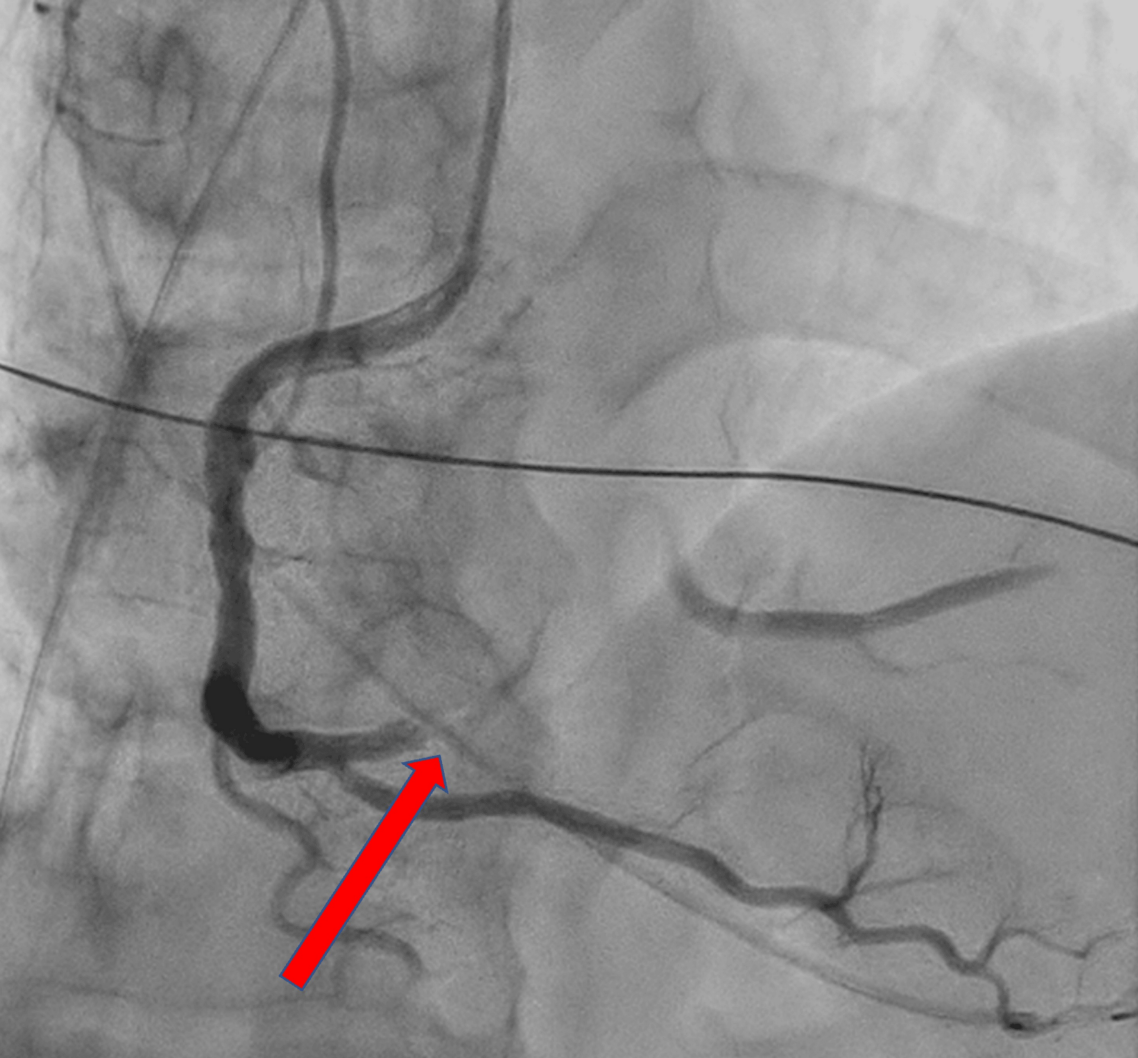
Emergency coronary angiography revealing severe stenosis of the proximal right coronary artery Emergency coronary angiography performed on August 27, 2025, at 12:30 PM revealed 99% stenosis with TIMI grade 1 flow in the proximal segment of the right coronary artery. The arrow indicates the site of severe stenosis. TIMI: thrombolysis in myocardial infarction.

**Figure 3:**
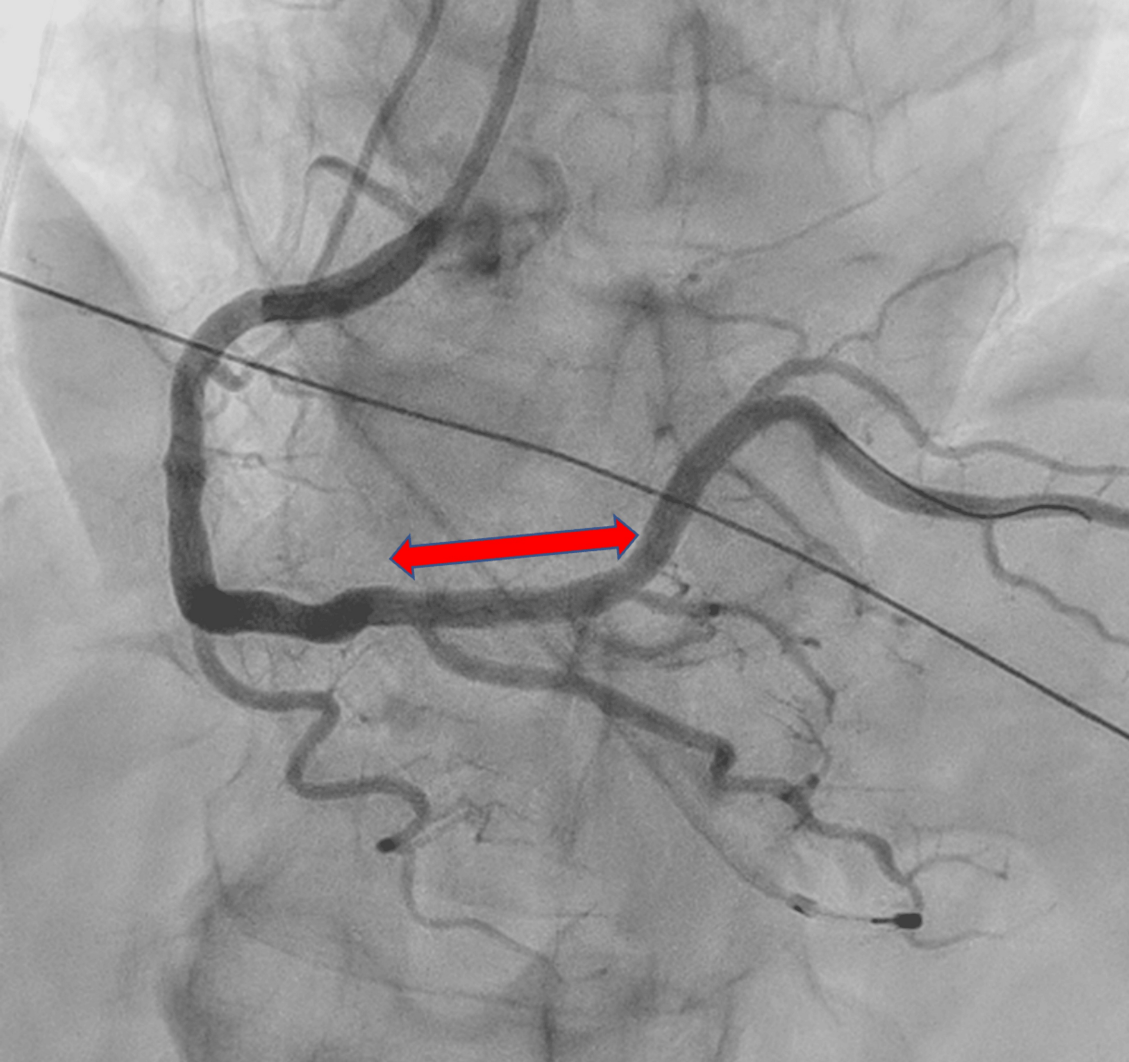
Post-intervention coronary angiography demonstrating successful stent deployment Post-intervention coronary angiography demonstrated successful percutaneous coronary intervention with deployment of a drug-eluting stent, restoring TIMI grade 3 flow in the right coronary artery. The arrow indicates the stented segment. TIMI: thrombolysis in myocardial infarction.

**Figure 4:**
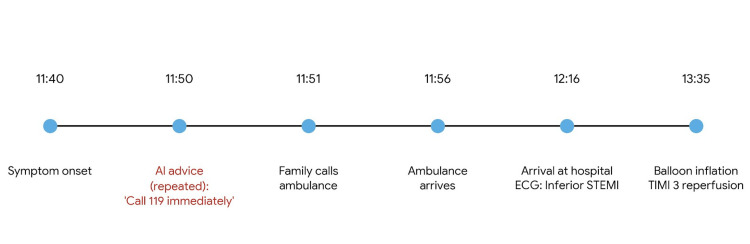
Timeline of clinical events from symptom onset to reperfusion Timeline of clinical events from symptom onset to reperfusion. Symptom onset occurred at 11:40 AM, AI-generated advice at approximately 11:45 AM, emergency medical services contact at 11:51 AM, hospital arrival at 12:16 PM, and successful reperfusion at 13:35 PM. Total symptom-to-balloon time: 115 minutes.

Emergency medical services transported the patient to a tertiary care hospital, where he arrived at 12:16 PM. A 12-lead electrocardiogram demonstrated ST-segment elevation in leads II, III, and aVF (arteriovenous fistula), consistent with acute inferior ST-elevation myocardial infarction (Figure 1) [2]. Emergency coronary angiography revealed a 99% stenosis with TIMI (thrombolysis in myocardial infarction) grade 1 flow in the proximal right coronary artery (Figure 2). Percutaneous coronary intervention was successfully performed with deployment of a drug-eluting stent, restoring TIMI grade 3 flow (Figure 3). A timeline summarizing the clinical course is shown in Figure 4.

Outcome and follow-up

**Figure 5:**
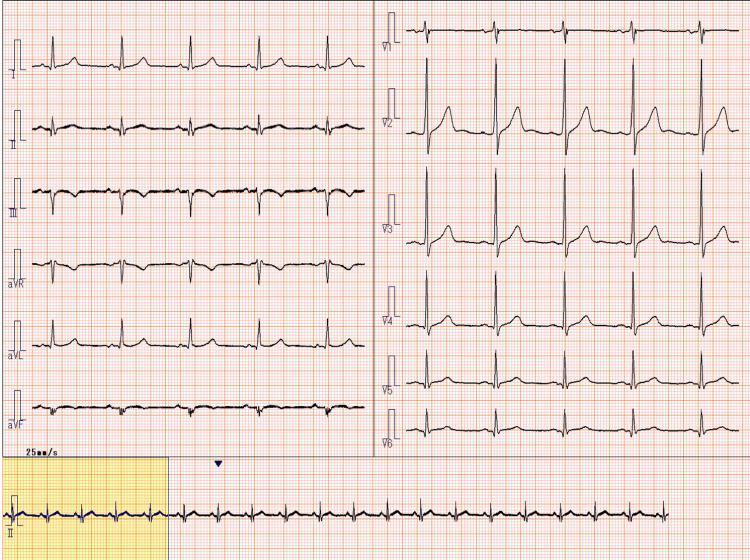
Follow-up electrocardiogram demonstrating resolution of ST-segment elevation Follow-up electrocardiogram obtained prior to discharge, demonstrating complete resolution of ST-segment elevation and return to baseline rhythm.

The total symptom-to-balloon time was 115 minutes, and the door-to-balloon time was 79 minutes, both of which were within guideline-recommended targets [2]. Peak creatine kinase reached 1,500 U/L, indicating a relatively limited extent of myocardial injury. The patient’s clinical course was uneventful. A follow-up electrocardiogram obtained prior to discharge demonstrated complete resolution of the ST-segment elevation (Figure 5).

## Discussion

This case demonstrates a distinct role for generative AI in acute care, functioning as a behavioral catalyst during the prehospital phase of illness. Importantly, this report does not evaluate diagnostic reasoning by AI, nor does it suggest that the patient relied on AI for medical diagnosis. The observed effect occurred at the level of behavior.

Reframing “typical” symptoms

From a clinical perspective, the patient’s symptoms were characteristic of AMI. However, this classification did not exist within the patient’s real-time cognitive process. The subjective prominence of toothache and the absence of dramatic vital sign abnormalities contributed to misinterpretation and delay. As noted in previous studies, craniofacial pain as the sole or primary symptom of AMI significantly increases the risk of missed diagnosis and death [6]. The AI did not identify a rare condition; rather, it objectively recognized a common but life-threatening pattern that the patient was cognitively discounting.

Linguistic imperative and behavioral action

Unlike conventional information sources that present probabilistic differentials, the AI employed an imperative speech act (“contact emergency medical services immediately”). This linguistic clarity reduced ambiguity and decisional inertia, transforming information into action. This phenomenon aligns with concepts in behavioral economics, where clear, actionable “nudges” can effectively alter health-related decision-making [9]. Furthermore, shared decision-making models typically involve clinician-patient interaction [10], but in the prehospital solitary phase, AI may serve as a surrogate partner to facilitate the decision to seek care. The clinical value observed lies not in diagnostic accuracy, but in the timing and clarity of the directive.

Scope and implications

Whether similar behavioral effects would be observed across different AI systems or languages warrants future investigation and lies beyond the scope of this case report. Nevertheless, in conditions such as acute coronary syndrome, where the cost of under-triage is high, AI-mediated over-triage may function as a beneficial safety net.

## Conclusions

Generative AI may contribute to improved outcomes in acute myocardial infarction by mitigating normalcy bias and accelerating patient decision-making. The benefit observed in this case arises not from enhanced diagnostic reasoning, but from the behavioral impact of clear, action-oriented language. As AI systems become increasingly accessible, their role in influencing patient behavior during the earliest phase of acute illness merits further study.
